# The ageing immune system as a potential target of senolytics

**DOI:** 10.1093/oxfimm/iqad004

**Published:** 2023-05-03

**Authors:** Peter Yandi Du, Ankesh Gandhi, Manraj Bawa, Justyna Gromala

**Affiliations:** Faculty of Medicine, Imperial College London, London SW7 2AZ, UK; Faculty of Medicine, Imperial College London, London SW7 2AZ, UK; Faculty of Medicine, Imperial College London, London SW7 2AZ, UK; Faculty of Medicine, Imperial College London, London SW7 2AZ, UK

**Keywords:** senolytic, senescence, immune system, ageing, therapy

## Abstract

Ageing leads to a sharp decline in immune function, precipitating the development of inflammatory conditions. The combined impact of these processes renders older individuals at greater risk of inflammatory and immune-related diseases, such as cancer and infections. This is compounded by reduced efficacy in interventions aiming to limit disease impact, for instance vaccines being less effective in elderly populations. This state of diminished cellular function is driven by cellular senescence, a process where cells undergo stable growth arrest following exposure to stressful stimuli, and the associated pro-inflammatory secretory phenotype. Removing harmful senescent cells (SnCs) using senolytic therapies is an emerging field holding promise for patient benefit. Current senolytics have been developed either to specifically target SnCs, or repurposed from cancer therapies or vaccination protocols. Herein, we discuss recent developments in senolytic therapies, focusing on how senolytics could be used to combat the age-associated diminution of the immune system. In particular, exploring how these drugs may be used to promote immunity in the elderly, and highlighting recent trials of senolytics in idiopathic pulmonary fibrosis and diabetic kidney disease. Novel immunotherapeutic approaches including chimeric antigen receptor T-cells or monoclonal antibodies targeting SnCs are being investigated to combat the shortcomings of current senolytics and their adverse effects. The flexible nature of senolytic treatment modalities and their efficacy in safely removing harmful SnCs could have great potential to promote healthy immune function in ageing populations.

## Graphical Abstract

**Graphical Abstract:**
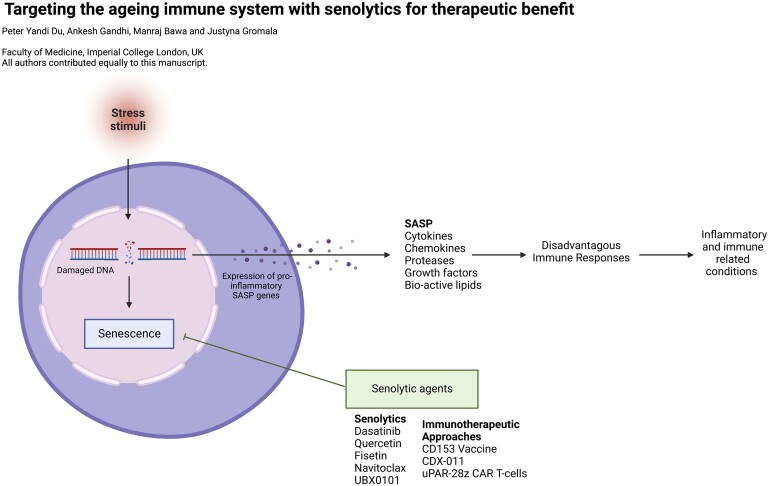

## Introduction

Age-related diseases account for approximately half of the current global disease burden [1], with poor health at old age associated with an increased risk of mortality. In addition, it is predicted that the elderly population will triple by the year 2050, which looks to increase stress on health and social care systems [2]. A growing body of evidence now links the development of many age-related diseases to senescence of the immune system [2].

**Figure 1.**
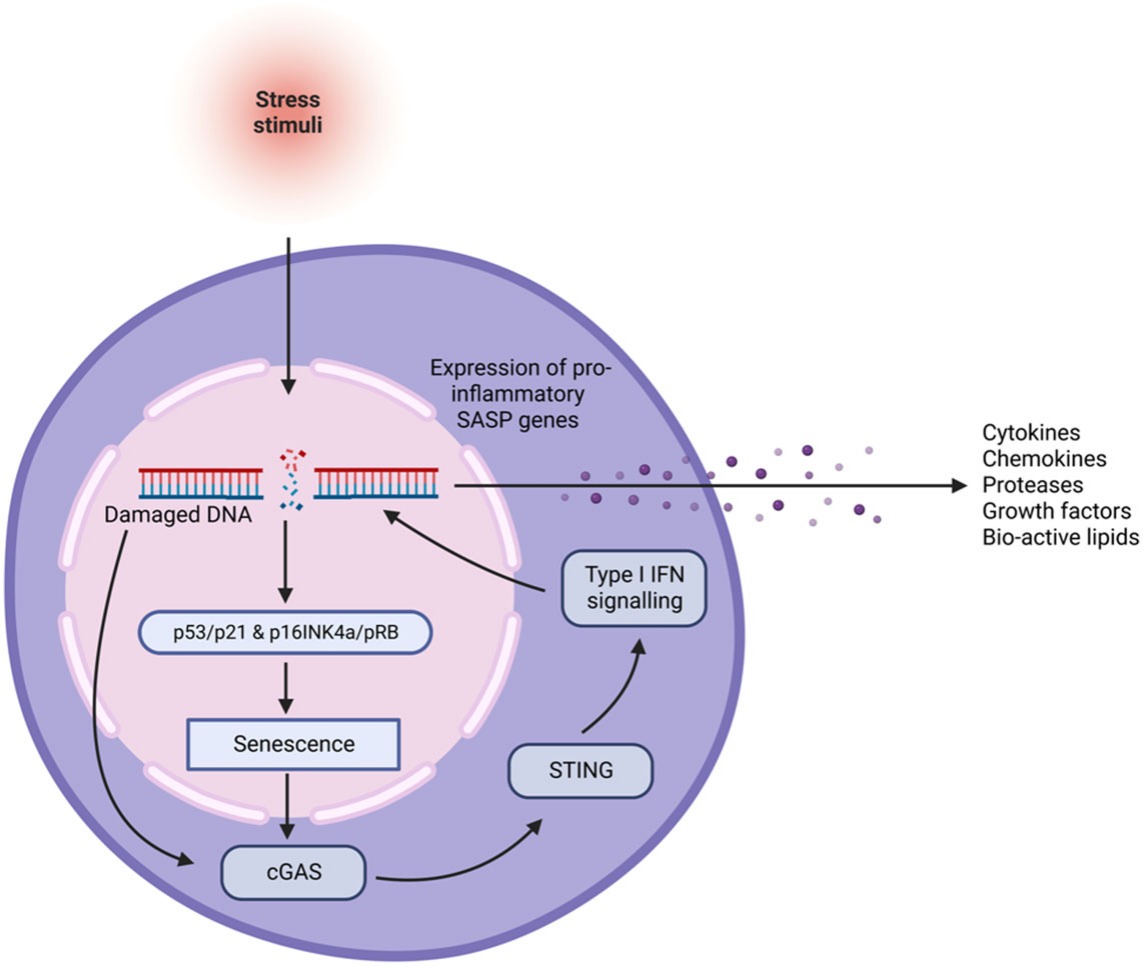
Cellular pathway of senescence and SASP development. The cGAS/STING pathway is one of several proposed mechanisms involved in SASP propagation. Cellular stress stimuli, for instance telomere shortening, ionizing radiation or oxidative agents, result in DNA damage. This leads to any one of the cellular senescence pathways (p53/p21 and p16INK4a/pRB pathways) being activated. Once a cell becomes senescent, damaged DNA can enter the cGAS/STING pathway which results in Type1 IFN signalling. This process drives the expression of the SASP, which produces pro-inflammatory mediators. Created using BioRender.

Cellular senescence is a process whereby cells experience stable growth arrest and phenotypic alterations through exposure to stimuli which cause DNA damage, for example, telomere shortening. It is thought this leads to prolonged activation of p53/p21 and p16INK4a/retinoblastoma protein (pRB) pathways which induce cellular senescence [3]. In the presence of cellular DNA damage, senescent cells (SnCs) have been shown to express pro-inflammatory proteins in a process termed senescence-associated secretory phenotype (SASP). Induced when DNA fragments bind to innate immune receptors causing downstream activation of the cyclic GMP-AMP synthase–stimulator of interferon (IFN) genes (cGAS/STING) pathway, triggering Type I IFN signalling [4]. This signalling, when paired with downregulation of p53 via the ubiquitin ligase SCF-Fbxo22 [5], is a crucial driver for SASP induction. SASP is characterized by its pro-inflammatory secretome, that releases various cytokines, chemokines, proteases, growth factors and bio-active lipids [3] (Fig. 1). Production of these inflammatory mediators is regulated through transcription factors, for example, nuclear factor (NF)-kB [6, 7].

The most prominent SASP component is interleukin (IL)-6, a pro-inflammatory cytokine linked to senescence pathogenesis. IL-6 primarily activates the JAK/STAT pathway, which is involved in modulation of cell cycle progression [8]. Other cytokines are also overexpressed in senescence, such as IL-8/C-X-C chemokine ligand-8 or IL-1: the latter of these influences NF-κB pathways, reinforcing the SASP [9]. These cellular processes are associated with age-related deterioration, and the collective impact of these processes on the whole organism is termed senescence. This is distinct from cellular senescence which refers to irreversible cell cycle arrest.

It is important to recognize that cellular senescence has an important beneficial physiological role in the recruitment of immune cells and regeneration of damaged tissue in the senescence-clearance-regeneration model [10, 11]. However, over extended periods, they can become risk factors for age-related diseases, such as idiopathic pulmonary fibrosis (IPF) or various presentations of arthritis [12–14]. Over time, SnCs accumulate due to poor immune clearance and their characteristic resistance to apoptosis [15, 16].

The immune system is essential in protecting the body from various pathogenic mechanisms and infection, in particular, there are significant changes to immune function as we age. Recent studies have suggested that the cellular senescence of immune cells impairs clearance of pathogenic material, increasing the risk of severe infections and mortality [17]. These processes lead to the build-up of inflammatory mediators, causing inflammageing—a state of chronic immune activation that is associated with blunted innate and adaptive immune responses [18]. The cumulative effect of these processes triggers downregulation of immune responses, via mechanisms such as defective lymphocyte responses and a reduction in regulatory immune cells [18]. Thus leading to deterioration of the immune system with age, termed immunosenescence. Thus, clearance of senescent immune cells could be beneficial to the immune system, as has been witnessed in other tissues [19–21]. This has driven research into modalities which can delay, or reverse, these age-related immune changes.

With greater life expectancy, there has been increased attention towards the use of senolytics in clinical settings. Current senolytic drugs aim to selectively apoptose SnCs, primarily through the inhibition of SnC anti-apoptotic pathway components. These include a range of protein-kinases—such as ephrin-B and phosphoinositide-3 kinases (PI3K)—as well as the B-cell lymphoma-2 (BCL-2) family of regulatory proteins, which encompasses BCL-2 itself, B-cell lymphoma-extra-large (BCL-xL) and B-cell lymphoma-w (BCL-w) (Table 1). The majority of current senolytic drugs are repurposed cancer therapies, for example dasatinib [25], with other novel techniques in preliminary research involving chimeric antigen receptor (CAR) T-cells, cardiac glycosides and caloric restriction [26–29].

**Table 1.** Examples of senolytics and their proposed mechanism of action

Senolytic	Senolytic mechanism	Type of SnC targeted	Developmental origin	Ref.
Dasatinib	Ephrin-B inhibitor	Preadipocytes Lung fibroblasts^a^	Anti-cancer drug	[21]
Quercetin	BCL-2 family inhibitor PI3K inhibitor	HUVECs Lung fibroblasts^a^	Naturally occurring flavonoid	[21, 22]
Fisetin	BCL-2 family inhibitor PI3K inhibitor	HUVECs	Naturally occurring flavonoid	[23]
Navitoclax	Pan-BCL-2 family inhibitor	HUVECs	Anti-cancer drug	[24]
UBX0101	p53/MDM2 interaction inhibitor	Osteoarthritic chondrocytes	Intentionally designed senolytic	[20]

a Combined therapy with D+Q targets senescent lung fibroblasts. HUVECs, human umbilical vein endothelial cells; p53, tumour protein 53; MDM2, mouse double minute 2 homolog.

Promising results have already been reported with use of dasatinib & quercetin (D + Q) in diabetic kidney disease [30] and IPF [31, 32]; both trialled in humans and mice. Moreover, a recent study investigating the use of senolytic approaches in mice infected with a beta-coronavirus analogous to severe acute respiratory syndrome coronavirus 2 (SARS-CoV-2) in humans [33]. A preliminary murine study shows that targeting SnCs could promote healthy ageing and increase lifespan [34]. Herein, we explore the impact of forthcoming trials whilst further discussing the potential pitfalls of current senolytic therapies and their potential application in infection.

## The current clinical landscape for senolytics

Ample pre-clinical evidence suggests that the clearance of SnCs can rejuvenate damaged tissues in various age-related diseases [23, 35–37]. Murine models thus far have been instrumental in the identification of novel senolytic drugs and drug targets. However, to date, clinical trials investigating senolytics have only been completed on several occasions [30, 32, 38] (Table 2), although there are additional studies currently in recruitment phases [39–41].

**Table 2.** Findings from clinical trials investigating senolytic drug activity

Study	Study type	Disease	Drugs/compounds investigated	Number of patient	Major findings	Authors	ClinicalTrials.gov identifier
Targeting pro-inflammatory cells in IPF: a human trial (IPF)	Single-armed open-labelled pilot study	IPF	Dasatinib +Quercetin	14	Beneficial effects to patient physical function, although improvements in other areas including lung function were not seen	Justice et al. (2019)	NCT02874989
Senescence in chronic kidney disease	Single-armed open-labelled pilot study	Chronic kidney disease	Dasatinib + Quercetin	9	Reduction in SnC burden and SASP factors	Hickson et al. (2019)	NCT02848131
A study to assess the safety and efficacy of a single dose of UBX0101 in patients with osteoarthritis of the knee	Phase II randomized controlled trial	Osteoarthritis	UBX0101	177	UBX0101 was not found to improve pain compared to placebo controls	Unity Biotechnology (2020)	NCT04129944

The senolytic combination of D + Q was first investigated in a single-armed, open-labelled pilot study for the treatment of IPF [32]. D + Q was intermittently administered to patients (*n *= 14) over three consecutive days in 3 weeks. At 1 and 2 weeks following treatment, a moderate beneficial effect was seen with regards to physical function, as assessed through the participants’ performance in various exercises. Furthermore, these improvements were seen 5 days after drug administration. This could suggest the potential for sustained benefits of senolytics beyond the treatment period, although this is yet to be conclusively demonstrated. Importantly, 12 of the 14 patients investigated regularly underwent these exercises, reducing the likelihood of a learned effect. However, critically, there were no improvements seen with pulmonary function, disease-specific health-related quality of life or circulating SASP components. Given that no changes were measured in SASP components, it remains to be questioned whether the beneficial aspects of D + Q were through senolytic changes or through alternative mechanisms.

The senolytic activity of D + Q has also been investigated in the context of diabetic kidney disease, using a similar single-armed open-labelled study design [30]. Patients (*n* = 9) were administered a 3-day oral treatment of D + Q with subsequent evaluation of SnCs and SASP components changes. The authors demonstrated the drug combination significantly reduced the SnC burden and SASP factors in patients eleven days following treatment.

It is important, however, that these results be interpreted with caution; these remain pilot-studies providing early proof-of-concept evidence for the potential of senolytics as therapeutics for age-related diseases. Critically, both aforementioned studies fail to include placebo controls and have limited sample sizes which culminates to limit the reliability of these findings [30, 32]. Additionally, while it was seen that the drugs have been capable of removing SnCs in murine models, it has yet to be seen how this clinically translates [23, 35–37]. Furthermore, the full spectrum of adverse events from SnC removal has yet to be fully realized and will need to be understood prior to clinical usage. Ultimately, larger, adequately powered and well-designed double-blinded studies are required to fully evaluate the clinical potential of these senolytics.

There is also interest in developing novel senolytic drugs as opposed to repurposing existing drugs. Primarily, these novel drugs would target the SnC apoptotic pathway and selectively drive SnCs towards apoptosis [36]. Of note, the senolytic UBX0101 was recently investigated as treatment for osteoarthritis reaching phase II clinical trials [42]. UBX0101 specifically targets the p53/MDM2 interaction and was found in pre-clinical mouse studies to reduce the number of SnCs in osteoarthritic joints [20]. Initially in phase I trials, UBX0101 was found to reduce knee pain based on a numerical rating scale, however, similar reductions in pain was not seen in the WOMAC—a more standardized assessment of pain and function in osteoarthritis [38, 42, 43]. In a subsequent phase II trial, a single dose of UBX0101 was given to 177 patients with osteoarthritis, with changes in the same pain scores being evaluated. The results of this trial were, however, discouraging as they indicated no significant improvement to pain or function between UBX0101 and placebo [38]. Furthermore, whether UBX0101 altered the expression of the SASP or the presence of SnCs in humans is unknown. Interestingly, other molecules that target the p53/MDM2 interaction were found to attenuate the SASP, rather than remove SnCs, which may contribute to a weaker senolytic effect than expected [44]. This would suggest that a single dose, as was given in the study, may not accurately portray the full effects of UBX0101. Furthermore, the placebo control used has been shown to improve pain in patients with osteoarthritis, and thus it has been argued that this could obstruct clinical findings [45]. Although progress with UBX0101 appears to have stalled, other novel senolytics are being investigated, for instance heat shock protein 90 (HSP90) or FOXO4-TP53 inhibitors [46, 47].

Although several clinical trials have been performed investigating the potential of senolytics, the results thus far have not identified a senolytic drug demonstrating a clear clinical benefit, although other senolytics are being investigated [39–41]. Additionally, it is important to emphasize that many of these trials are early-stage clinical trials, and so drawing conclusions on the potential of senolytics as a whole may be premature. Nevertheless, investigating and developing novel senolytics that target new pathways could lead to improved outcomes over current senolytics and provide a greater clinical benefit.

## Senolytics—targeting senescent immune cells

Current senolytics aim to clear all SnCs, regardless of their cell type, however, refining this to target specific SnC populations may be of benefit, by increasing the efficacy of drugs whilst reducing potential side effects, as seen in other fields such as cancer therapies [48]. One of the major concerns of an ageing immune system is its diminished immune response, which in part is driven by senescent immune cells [17, 18]. This effect of an ageing immune system can be seen clinically, as older populations are at greater risk of succumbing to serious infections and have poorer efficacies with regards to vaccines. Senolytics targeting senescent immune cells may provide a solution to help improve immunity within elderly populations [19–21].

**Figure 2.**
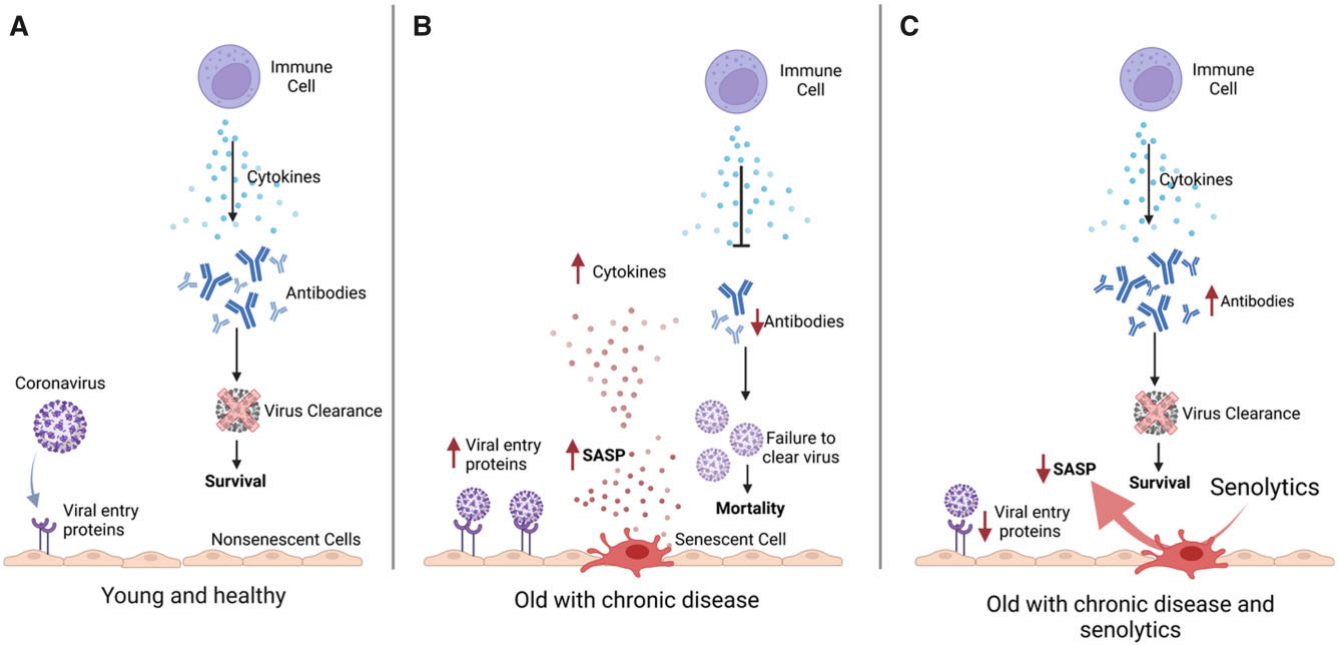
The impact of SnCs on immune response to a coronavirus in murine models and effects of using senolytics. A, The absence of SnCs in young healthy individuals leads to an appropriate inflammatory response and viral clearance. B, Aged individuals with chronic disease have more SnCs that create a more pro-inflammatory environment at baseline. SnCs exacerbate this pro-inflammatory environment, impair viral clearance and are attributable to worse outcomes including higher mortality following coronavirus infection in mice. C, Senolytics can rejuvenate the immune response and aid viral clearance by decreasing inflammation, SnCs, mortality and improving the antibody response. Figure adapted from Camell *et al.* [30] using BioRender.

Looking specifically at coronavirus disease-19 (COVID-19) older age was identified as a key risk factor for fatal outcomes [49, 50]. Drugs proposed for treating COVID-19 early in the pandemic, including azithromycin, quercetin and chloroquine-related compounds, have senolytic properties [49]. A recent study found that human endothelial SnCs generate hyperinflammatory responses to pathogen-associated molecular patterns, including the S1 spike protein of SARS-CoV-2 [33]. Using animal models, the authors also showed that SnCs in the elderly are accountable for worse outcomes in infections (Fig. 2). Treating older mice with either fisetin, D + Q or genetic ablation reduced mortality following exposure to common pathogens by approximately 50% compared to untreated mice. An important caveat to experiments with mouse models in this study is that the authors infected mice with mouse hepatitis virus A59, a β-coronavirus related to SARS-CoV-2. Whilst this is a useful model, using a different species to humans and a virus that is not SARS-CoV-2 may not accurately reflect human physiology and response to SARS-CoV-2 infection. The group also proposed the ‘Inflammation Amplifier/Rheostat Hypothesis’, wherein higher baseline inflammation and an amplified response to pathogens, compared to environments without SnCs, leads to increased infection-driven pathology and thus a more severe course of disease [33]. SARS-CoV-2 has been shown to induce cellular senescence in previously non-SnCs, where the S1 surface protein of SARS-CoV-2 enhanced SASP in cultured human cells [51], highlighting similarities in SASP generation and its impacts in both humans and mice. In mice and hamsters infected with SARS-CoV-2, the senolytics navitoclax and D + Q removed virus-induced senescence, reduced inflammation as well as lung disease [52].

Administration of quercetin as an adjuvant to standard care in the early stages of the disease course has been shown to have several favourable effects in a cohort of 152 people that developed COVID-19, including lower frequency and duration of hospital stays, fewer deaths, as well as a possible anti-fatigue and pro-appetite effect with a good safety profile [53]. The study has limitations, including the lack of blinding and a relatively small cohort, but warrants resource allocation to future trials with placebo and blinding to assess reproducibility of results. Another pilot study also compared individuals with COVID-19 receiving either standard of care treatment alone or with quercetin [54]. The latter group showed faster improvement in symptoms, quantitative reverse transcription polymerase chain reaction (PCR) test conversion from positive to negative and reduced blood levels of markers including C-reactive protein and D-dimer [54]. However, it is noteworthy that the treatment arm was significantly younger than the control arm which may confound results. The COVID-FIS trial assessing the use of fisetin in elderly skilled nursing facility residents that become PCR-positive for SARS-CoV-2 infection, is currently ongoing [55].

To further understand the impacts of senescence on immune function and the potential of senolytics, a study assessed responses to intranasal H1N1 influenza infection in C57BL/6 mice [56]. This work suggests that SnCs impact T-cell differentiation, promoting the differentiation and accumulation of Forkhead box 3 (Foxp3)+ regulatory T-cells (Tregs) in tissues. Tregs are found in higher proportions in lungs of old mice compared to young mice. Higher levels of TGF-β were also found in bronchoalveolar lavage (BAL) of old mice, underlining the fact that transforming growth factor (TGF)-β promotes FoxP3 expression [57]. Blocking TGF-β consequently led to a reduction in FoxP3-expressing cells. Since SnCs are known to express TGF-β, it is possible that SnCs promote the differentiation of Tregs in tissues. The authors tested this by treating mice with D + Q and then exposing them to a sublethal influenza infection model, showing that this treatment modified the immune response of aged mice to resemble that of a younger mouse. This featured a reduction in FoxP3 expressing cells and T-cell differentiation towards a T-helper cell type 2 (Th2) phenotype, associated with healing and restoration of homoeostasis. Importantly, D + Q reduced the concentration of TGF-β in BAL, suggesting that SnCs contribute to TGF-β production, driving the accumulation of Tregs in the airway and potentially impairing immune response to infection. Future studies should determine if this is also seen in humans. Further research examining effects of senolytics on the immune response towards other viral and non-viral pathogens would determine whether targeting SnCs is a viable therapeutic target in this clinical setting.

It is important to note that these studies were often early-stage studies and had their limitations in study design. Furthermore, several studies have identified senolytic properties from non-pharmacological interventions including calorie restriction and exercise, as excellently reviewed by Fontana *et al.* [29]. None of the studies mentioned herein reported lifestyle factors in their participants, highlighting this potential confounder to be addressed in future work. Despite these limitations senolytics could impact future management of infections in the elderly; for example, senolytics could protect this vulnerable group in the future, limiting the impact of infections, as seen with the COVID-19 pandemic. The positive findings of these early studies warrant gaining a better insight into how useful, safe and cost-effective senolytics could be as when used in larger cohorts.

Targeting senescent immune cells has further drawn interest in the treatment of age-related diseases. Recent studies have implicated senescent immune cells in driving senescence and ageing in tissues including the liver in mouse and rat models [58, 59], although the precise mechanism behind this is yet to be determined. Given these findings, it is tempting to speculate that senescent immune cells are driving similar age-related changes in other tissues and organs. Targeting senescent immune cells therapeutically could therefore provide benefits beyond that of targeting individual tissue-based SnC populations. So far, a mouse model has demonstrated that neutralization of a key SASP component from senescent immune cells using monoclonal antibodies could alleviate progression of age-related skeletal changes [59]. Investigating whether this same attenuation of the SASP from senescent immune cells can simultaneously improve outcomes for other age-related diseases could therefore be of interest.

Although current senolytics may have some degree of senescent immune cell clearance, a senolytic specifically targeting senescent immune cells has not yet been developed. Given the impact of senescent immune cells on the pathophysiology of age-related disease and the diminution of the immune response, senolytics targeting these cells may have multi-faceted benefits. Additionally, whilst research is still in early stages, pre-clinical models show that senolytics could modulate a more favourable immune response to infection in older mice and humans [33, 52].

## The flip side of senolytics: challenges to overcome

The conceptual promise of using senolytics to both target the ageing immune system and manage infection is clear, yet there remain key limitations and uncertainties that current and future senolytics must address. For instance, despite an undoubted association with numerous deleterious effects, senescence has also proven central to certain homoeostatic mechanisms. There is a paucity of literature investigating how the removal of SnCs will impact upon such mechanisms in humans, and to what extent these disruptions on a cellular level will result in clinical toxicities.

The beneficial role of senescence and SnCs is most well-defined in tissue remodelling and repair. In mouse models, senescent fibroblasts have been found to secrete the SASP factor, platelet-derived growth factor AA [60], which promoted myofibroblast differentiation and granulation, accelerating wound repair. Similarly, the extracellular matrix protein Cysteine rich 61 (CCN1) could induce senescence in both cutaneous and hepatic fibroblasts in mice [61, 62]: this limited fibrosis during wound repair and following chronic liver injury, respectively. Another mouse model of keratinocytes demonstrated that transient exposure to SASP products induced cellular plasticity in neighbouring cells, promoting tissue regeneration [11]. Importantly, however, this beneficial effect was observed only with limited SASP exposure: prolonged exposure along with aberrant SnC clearance was associated with tumourigenesis.

Tumourigenesis is a potential harmful effect of chronic SnCs, which can accumulate in ageing due to inadequate clearance [63], but senescence itself also plays crucial roles in tumour suppression. Acute, transient SnCs with oncogene mutations induce cell cycle arrest and enhance immunosurveillance [64, 65]. Further, seemingly contradictory, effects of senescence are seen in other pathologies. Fibroblast senescence following myocardial infarction enhances cardiac repair [66], but a separate mouse model demonstrated that SnC clearance by the senolytic navitoclax prolonged survival post-myocardial infarction [67]. Senescent pancreatic beta cells have also been observed to increase insulin secretion, and clearance of these cells may consequently precipitate a dysregulation in glucose homoeostasis [68]. It is important to note that the evidence behind the benefits of senescence is largely derived from mouse models, which may not fully reflect the complexities of senescence and its role in pathophysiological processes in humans. Nonetheless, it is apparent that using senolytics to either clear senescent immune cells directly or enhance immune clearance of SnCs entails an inherent risk of limiting the protective impacts of senescence. Until further research is conducted to robustly examine how this risk translates into clinical complications, the real-world applications of senolytics are restricted significantly.

There is also a degree of contrasting evidence on the role of senolytics in anti-viral immunity. Senolytics have demonstrated promise in downregulating viral replication and even clearing SnCs infected by a wide range of viruses *in vitro* [69, 70]. Conversely, the use of dasatinib significantly increased the subsequent risk of cytomegalovirus reactivation in a cohort of leukaemia patients [71]. Importantly, this study examined the effect of dasatinib following haematopoietic stem cell transplant, allowing its direct impact on the immune system to be evaluated. Dasatinib has also been linked to the reactivation of other viruses in leukaemia patients, including hepatitis B virus [72], though evidence for this association is weaker and limited to very few cases. Encouragingly, other senolytics, such as quercetin, fisetin and navitoclax, have not been associated with viral reactivation [69]. This indicates that the viral reactivation effect is likely specific to dasatinib, as it inhibits a wide range of tyrosine kinases which regulate immune function [73]. Current and future senolytics with different mechanisms-of-action should eliminate this problem.

Furthermore, there is concern about potential ‘off-target’ toxicities of existing senolytics against non-SnCs. D+Q have both demonstrated adverse effects in humans [32], with the former particularly associated with fluid retention and myelosuppression [74], although these are generally considered to be moderate, controllable or reversible upon discontinuation [30, 70]. Both dasatinib and navitoclax have been associated with thrombocytopaenia in previous clinical trials [75, 76]. Navitoclax targets and inhibits BCL-xL, an anti-apoptotic protein in SnCs that also regulates platelet survival; navitoclax treatment led to grade 3/4 thrombocytopaenia in 29 of 55 lymphoid malignancy patients in an earlier phase I clinical trial [77]. Although clinical manifestations of this thrombocytopaenia were limited to only one instance of bleeding, this remains a worrying example of off-target toxicity.

Navitoclax also exhibited cytotoxicity against osteoblasts and bone marrow progenitor cells in mice through a currently unknown mechanism, resulting in a significant loss of bone mass [78]. Moreover, novel senolytics such as HSP90 inhibitors and macrolide antibiotics have demonstrated varying degrees of activity against non-SnCs *in vitro* [47, 79]. Future senolytics with different cellular targets may also cause on- or off-target toxicities to other cell types which are not affected by current senolytics. It can therefore be speculated that as yet unidentified adverse effects of senolytics against non-SnCs may emerge. These could be similar in nature to the autoimmune toxicities demonstrated by immunotherapies such as chimeric antigen receptor T-cell (CAR T-cells) and checkpoint inhibitors [80, 81]. Based on this possibility, and the demonstration of toxicities in mouse models, clinical trials of current and future senolytics are required to assess how toxicities might manifest in humans.

Novel strategies aimed at counteracting such senolytic-associated toxicities are emerging. Given that current markers of senescence are not specific to SnCs [82, 83], this further poses a challenge in precisely identifying SnCs and quantifying their pathological burden. Identifying a unique marker for SnCs would not only clarify the role of SnCs in disease pathogenesis but also potentially enhance the selectivity of senolytics. This could in turn reduce the risk of off-target adverse effects. Although not perfect, the characterization of cellular senescence by senescence-associated (SA) β-galactosidase activity has proven useful in this respect [84]. Activity of SA-β-galactosidase led to discovery of CD26 as a surface protein that could be a drug target against SnCs [85], where CD26 expression was greater in elderly patients’ peripheral blood mononuclear cells compared with younger individuals. Therefore, CD26 could potentially be used as a specific target for senescent immune cells. Furthermore, antibodies against CD26 have been successfully generated to selectively remove SnCs in *in vitro* models, via antibody-dependent cell-mediated cytotoxicity [85]. In fact, anti-CD26 antibodies have already been demonstrated to selectively target CD26-expressing cells in mouse models for cancer immunotherapies [86]. Determining whether CD26-targeting antibodies can clear senescent immune cells *in vivo* would highlight the translational potential of such an approach.

Senolytics could also be modified on an individual basis to limit adverse effects; this principle has already been demonstrated to reduce the anti-platelet toxicity of navitoclax in a preclinical study [87]. However, such strategies are only feasible if the core senolytic activity of the drug is not significantly compromised through modification. Nonetheless, it is clear that potential methods to minimize the established or theoretical adverse effects of senolytics do exist. These strategies require further investigation but may yet prove an important step towards maximizing the practical applications of current and future senolytics.

## Immunotherapeutic approaches to senolysis: an ambitious crossover?

Immunotherapies could overcome challenges experienced with current senolytics. The role of vaccines in removal of senescent T-cells has been examined in utilizing a CD153 vaccine in mice, which showed a reduction in SnCs [88]. However, once again, absence of an adequate senescence marker poses a challenge in evaluating these results. Other proposed targets for a neutralizing senolytic vaccines are beta-2 microglobulin and C-C chemokine ligand (CCL) 11, factors found in the blood that are thought to be pro-ageing [89]. A study identified glycoprotein nonmetastatic melanoma protein B (Gpnmb) as another target for senolytic therapy [90]. Gpnmb is expressed, for example, in vascular endothelial cells of patients with atherosclerosis, but not in those without the disease. The authors trialled a vaccine that eliminates cells expressing Gpnmb and found that this improved atherogenesis caused by high-fat diet and metabolic dysfunction in mice [90]. Because Gpnmb also has physiological roles, monoclonal antibody therapy has been explored [91]. The anti-Gpnmb monoclonal antibody CDX-011 (glembatumumab vedotin), has been used clinically in melanoma and triple negative breast cancer treatment. While this antibody did not display sufficient anti-tumour activity to extend survival, it could be repurposed to target SnCs. Newer antibody technologies could also be repurposed for senolytic therapies. Bi-specific antibodies could be explored to target both a marker of senescence and an immune cell marker to improve specificity of treatment [92, 93]. Alternatively, antibody-drug conjugates could similarly be investigated to deliver senolytics towards SnCs, in an attempt to localize drug actions [94, 95].

A major impact of ageing on the immune system culminates in poor responsiveness to vaccines, perhaps best exemplified in BioNTech SARS-CoV-2 vaccination, where older persons had significantly lower SARS-CoV-2 antibody titres and specific T-cell reactivity [33, 96, 97]. To date, the majority of methods that aim to improve vaccine responses in older people have either increased antigen dose or added adjuvants to stimulate the immune response [98]. It has been suggested that inhibiting the SASP may generate superior production of influenza antibodies [33, 99]. Giving patients a senolytic before vaccination might have the same effect and thus improve vaccine responses in older people. While it remains to be seen whether such an approach would have a clinical benefit, the idea is an exciting hypothesis.

Recent studies have evaluated the role of CAR T-cells in targeting SnCs. Of great relevance is a proof-of-principle study by Amor *et al.* [100]. The team identified urokinase-type plasminogen activator receptor (uPAR), which is also of interest in inflammatory conditions and certain cancers, as a target highly expressed by SnCs. The group developed uPAR-28z CAR T-cells that eliminated SnCs and led to a reduction in liver fibrosis and damage in mice. However, uPAR can also be found at low levels in healthy tissues, including bronchial epithelium. Therefore, careful safety evaluation is needed during further investigation of SnC-targeting CAR T-cells as an alternative senolytic modality programming the immune system to target senescence.

Overall, novel immunological strategies, including vaccines, CAR T-cells and monoclonal antibodies offer alternative senolytic strategies. They come with their own challenges and possible risks but may also hold the potential to ameliorate certain limitations observed in current pharmacological approaches. These come with their own challenges and possible risks but may also hold the potential to ameliorate certain limitations observed in current pharmacological approaches.

## Conclusion

Cellular senescence involves the activation of key pathways, including the JAK/STAT, p53 and p16INK4a, in synergism with the SASP inflammatory secretome. With age, this impairs immune reactivity, and can also cause the development of inflammatory disorders. The growing elderly population justifies research into a new class of drugs targeting SnCs: senolytics.

To date, several clinical trials have investigated the role of senolytics in the treatment of age-related diseases and infections, including COVID-19. Whilst the results are promising, significant progress is required before senolytics are used routinely in clinical practice. There is much anticipation of the results of active and recruiting clinical trials to see what further promise senolytics hold. Additional interest has developed in targeting senescent immune cells with these senolytics, with potential drug targets and drug development strategies being investigated. However, using senolytics to enhance immune clearance of SnCs or to target senescent immune cells themselves may also have deleterious impacts. These range from inhibiting the beneficial effects of senescence to causing off-target toxicities. Novel strategies to overcome such challenges are emerging, including vaccines, CAR T-cells and monoclonal antibodies. Identifying target markers for senolytics that are entirely specific to SnCs will limit off-target toxicity, with CD26 holding promise in this regard.

Overall, there remains significant work to be done to determine safe and specific targets that will allow for targeted killing of SnCs. Large-scale clinical trials of senolytics are needed to fully assess their efficacy and safety profile in humans, both short and long terms. With sufficient recognition and appropriate fund allocation towards the field of senolytics, significant advances could be made in the way we treat infection and age-related diseases.

## Data Availability

Data sharing is not applicable as no new data has been presented here.
